# An update on clonality: what smooth muscle cell type makes up the atherosclerotic plaque?

**DOI:** 10.12688/f1000research.15994.1

**Published:** 2018-12-21

**Authors:** Stephen M. Schwartz, Renu Virmani, Mark W. Majesky

**Affiliations:** 1 Pathology, University of Washington, Seattle, WA, 98112, USA; 2 CV Path Institute, Gaithersberg, Maryland, 20878, USA; 3 Center for Developmental Biology and Regenerative Medicine, Seattle Children's Hospital Research Institute, Seattle, WA, 98112, USA

**Keywords:** Atherosclerosis, smooth muscle cells, developmental biology, vascular biology, clonality, somatic mosaicism, endothelium. neoplastia, cancer, myofibroblast, pericyte, fibroblast, mesoderm, intimal hyperpasia

## Abstract

Almost 50 years ago, Earl Benditt and his son John described the clonality of the atherosclerotic plaque. This led Benditt to propose that the atherosclerotic lesion was a smooth muscle neoplasm, similar to the leiomyomata seen in the uterus of most women. Although the observation of clonality has been confirmed many times, interest in the idea that atherosclerosis might be a form of neoplasia waned because of the clinical success of treatments for hyperlipemia and because animal models have made great progress in understanding how lipid accumulates in the plaque and may lead to plaque rupture.

Four advances have made it important to reconsider Benditt’s observations. First, we now know that clonality is a property of normal tissue development. Second, this is even true in the vessel wall, where we now know that formation of clonal patches in that wall is part of the development of smooth muscle cells that make up the tunica media of arteries. Third, we know that the intima, the “soil” for development of the human atherosclerotic lesion, develops before the fatty lesions appear. Fourth, while the cells comprising this intima have been called “smooth muscle cells”, we do not have a clear definition of cell type nor do we know if the initial accumulation is clonal.

As a result, Benditt’s hypothesis needs to be revisited in terms of changes in how we define smooth muscle cells and the quite distinct developmental origins of the cells that comprise the muscular coats of all arterial walls. Finally, since clonality of the lesions is real, the obvious questions are do these human tumors precede the development of atherosclerosis, how do the clones develop, what cell type gives rise to the clones, and in what ways do the clones provide the soil for development and natural history of atherosclerosis?

## Introduction

Although Earl Benditt and his son John described the clonality of the atherosclerotic plaque in 1973 ^1^ and although their observations have been confirmed many times ^2– 7^, interest in the idea that atherosclerosis might be a form of neoplasia waned because of the clinical success of treatments for hyperlipemia and because of the availability of transgenic mice as models for the effects of dysfunction of lipid metabolism on the vessel wall.

There are reasons to reconsider Benditt’s hypothesis. Over the last five years, the concept of clonality, both in cancer and in normal development, has changed. Over a longer period, we have also learned a great deal about the developmental biology of the normal vessel wall, including the normal formation of clonal patches in that wall and the development of a normal layer of smooth muscle cells (SMCs) in the intima long before atherosclerosis itself ^4, 8^. As a result, comparisons between atherosclerotic lesions and neoplasms now need to be rethought ^9^.

Benditt’s hypothesis also needs to be revisited in terms of changes in how we define SMCs. We now know that these cells derive from several different mesodermal precursors, and recently criteria for defining cell types have been changing as a result of new insights driven by the analysis of single-cell expressomes.

Finally, since clonality of the lesions is real, the obvious questions are do these human tumors precede the development of atherosclerosis, how do the clones develop, what cell type gives rise to the clones, and in what ways do the clones provide the soil for development and natural history of atherosclerosis?

## Does atherosclerosis begin with a neoplastic event?

Benditt’s 1973 paper ^10^ was, to use a term familiar from today’s tech industry, “disruptive”. The paper showed that atherosclerotic lesions were clonal cell masses in the atherosclerotic intima. Benditt suggested that because clonality was a mark of cancer, the lesions arise as benign neoplasms ^11^. This suggestion was based on an analogy to the benign uterine leiomyoma ^11– 20^. Like atherosclerotic plaques, uterine leiomyomas are monoclonal masses of SMCs. Like plaques, uterine leiomyomas are present in all humans, at least if “all” means most human females. Leiomyomas are made up of uterine smooth muscle. Using microdissection and immunocytochemistry, Benditt and Benditt identified the plaque clonal cell type with vascular SMCs. The team of Majesky, then a student working with Benditt Sr., went on to show that carcinogens, even without fat feeding, produced nodular smooth muscle tumors in the intima of chickens ^21^ and so clonality of masses in the intima can arise as a neoplastic event.

Although Benditt’s data were repeatedly confirmed in human lesions ^2– 7^, interest in a “neoplasia hypothesis” was trumped. The success of lipid-lowering drugs maintained a focus on the traditional lipid hypothesis. The ease of studying atherosclerosis in experimental animals, especially inbred mice with transgenic modifications in lipid metabolism, permitted extensive studies of the arterial response to accumulation of lipid, even though the murine models have not, as of yet, fully modelled the advanced lesions that cause human death ^22, 23^.

Nonetheless, Benditt was at least formally correct. Spontaneous intimal masses (that is, smooth muscle-like collections in the intima) fit the usual definition of a benign tumor. In humans, these masses appear in the arterial intima prior to lipid accumulation ^24, 25^. The obvious questions are how do the intimal cell masses develop and in what ways do intimal masses provide the soil for development and natural history of atherosclerosis?

**Figure 1.**
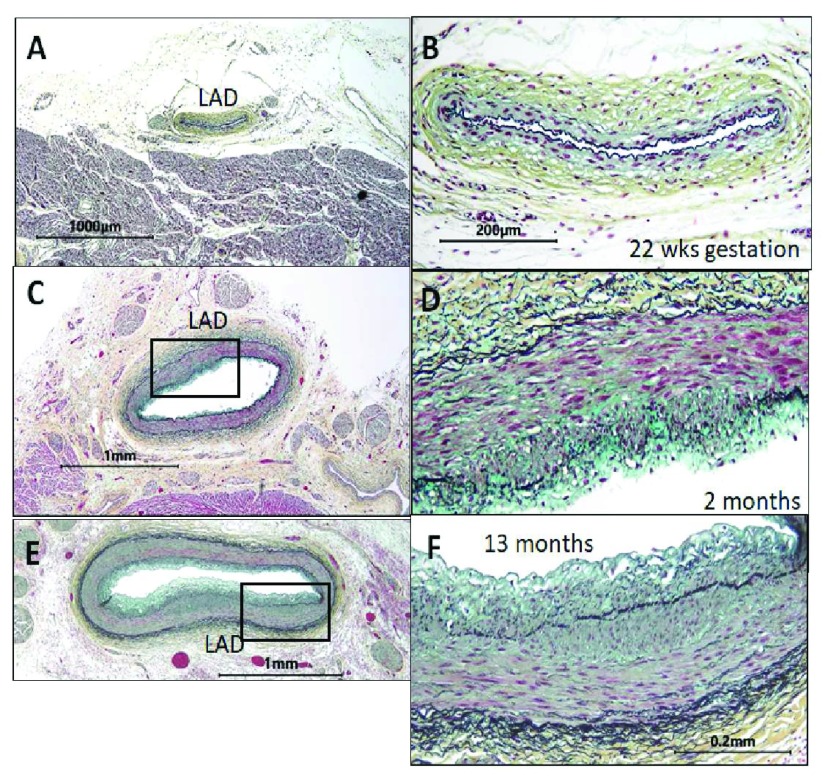
The normal intima occurs independently of and prior to the fat feeding associated with atherosclerosis ^4^. These images of the left anterior descending coronary artery (LAD) show the absence of cells in the intima *in utero*. The images to the right ( **B**, **D**, and **F**) are enlargements of the LAD arteries to the right ( **A**, **C**, and **E**). The endothelium at 22 weeks of gestation rests on the internal elastic lamina ( **A**, **B**). By 2 months ( **C**, **D**), a cellular intima is present in the LAD of this normal heart. By 13 months ( **E**, **F**), this intima is about 50% as thick as the underlying media (400×, Verhoeff–van Gieson’s stain). In adult vessels, these normal sites would have progressed to form atherosclerotic plaques.

Figure 1 shows intimal cell masses in the arteries of human infants. These masses appear during development prior to lipid accumulation ^24, 25^. Evidence that these sites are clinically important came from the classic PDAY (Pathobiological Determinants of Atherosclerosis in Youth) study ^26^. PDAY studied the arteries of young people who had died of non-cardiac causes. The most reproducible locations of advanced atherosclerotic lesions in these young people included the left descending coronary artery, the right coronary artery, the carotid bulb, and a region of the abdominal aorta below the diaphragm.

The sites identified in PDAY as being the most reproducible lesions are only a subset of the sites where fatty streaks, the initial lesions seen in fat-fed animals and in older human children, occur. Thus, atherosclerotic lesions can develop with and without pre-existing intimal cell masses. Moreover, there is no evidence that the development of intimal masses is necessary for the lipid-dependent processes leading to fatty streaks. Atherosclerotic diets fed to all mammals, including humans, result in accumulations of lipid- and fat-filled macrophages in the intima at arterial branch sites where blood flow is disturbed. Such sites include the openings of the intercostal arteries in fat-fed mice, rabbits, swine, or primates. Fatty streaks are also seen at branch points of thoracic arteries in young humans but recede as we age ^27, 28^. Further studies by DeBakey and Glaeser ^29^ used angiography to follow the occurrence of newly formed lesions over 25 years in a large number of patients who initially had documented occlusive atherosclerosis at specific sites. The location of the new lesions in different arterial beds did not fit any well-described hypothesis such as distribution of blood flow. Perhaps the location of lesions was due to pre-existing intimal cell masses. Since these may be the sites where clinically significant lesions occur, perhaps the animal models do not fully model the human disease. Perhaps development of the fully developed, clinically significant lesions in adult humans depends on the initial processes, the cellular “soil” giving rise to a lesion in childhood.

## Benditt versus Virchow

In Virchow’s seminal 1856 text, “Cellular pathology” ^30^, the founder of modern pathology did not look at these earliest lesions. Instead, Virchow hypothesized that the adult atherosclerotic lesions he saw were the result of an inflammatory process stimulated by the toxic products of lipid accumulating in the intima. Migration and then proliferation of the cells we now call medial SMCs were part of Virchow’s view of this response to injury. Over 150 years later, Virchow’s “response to injury hypothesis” ^31^ is supported by the ability of scientists to produce intimal lesions by feeding fat-rich diets to a variety of mammals, including mice, rabbits, pigs, and monkeys ^23, 32– 41^. The “lipid hypothesis” or “response to injury hypothesis” remains dominant because of the obvious fact that lipid feeding in animal models produces lesions even in mice that lack a normal intima ^42, 43^ and the fact that lipid-lowering drugs greatly decrease the incidence of death from atherosclerosis in humans ^44^.

It seems reasonable, based on studies in mice, to believe that medial SMCs are recruited into the intima to form a fibrous cap encapsulating the fatty accumulation ^45^. Cell tracing studies now show that this fibrous cap in mice is derived from medial SMCs ^8, 45– 49^. Moreover, recent studies show that this neointima, formed in response to the lipid-induced injury in mice, is clonal ^48, 50^. These cells surround lipid deposits and lipid-rich macrophages derived from blood monocytes formed much as proposed by Virchow. A huge amount has been learned about how the bone marrow-derived, fat-filled monocytes accumulate and enter the intima in response to inflammatory changes in endothelial cells induced by focal changes in blood flow or by hyperlipemia ^51^.

If the mouse model is correct, fatty lesions begin before the formation of an intimal cell mass. If this were true in humans, clonality of atherosclerotic lesions might be irrelevant. However, we know that intimal cell masses develop at critical sites before lipid accumulation and, as we will discuss below, we still do not know how these cell masses form or why they become clones. Moreover, even the identity of the cells comprising the clones is unclear. Benditt’s use of “smooth muscle” to identify the intimal cells may itself have been simplistic because the identification of all cells that coat vessels as “smooth muscle” is being challenged. Recent reports show that some (or perhaps many) of these “plaque macrophages” are derived from medial SMCs that have assumed a macrophage phenotype ^49, 52– 55^. Other studies raise issues about the use of smooth muscle alpha actin to define the SMC type. For example, recent studies show that the mural cells that form the coat around certain capillaries in the brain lack smooth muscle alpha actin ^56^. Even if cells making up the vessel wall express smooth muscle markers ( Table 1), cells comprising the mural cell coats have embryological origins from different regions of the mesoderm ^46, 57– 61^. Thus, diverse “smooth muscle” subtypes that could give rise to the intima and diverse phenotypes could be important to the natural history and long-term outcome of the lesions.

**Table 1.** Mural cells, cell types that make up arterial wall coats around the endothelium.

		Marker genes	References
Medial smooth muscle cells	Typical coats seen around endothelial and endodermal tubes. Usually called “smooth muscle” but, as discussed in the text, cells lacking actin-rich contractile proteins have also been seen in the media.	Smooth muscle alpha actin, smooth muscle myosin, smooth muscle 22α, calponin, and desmin	62– 110
Intimal cells	Cells located between the endothelium and the medial layers. Under endodermally derived epithelium, these cells are called the “lamina propria”.	See text. In general, intimal cells have been described as having markers shared with smooth muscle cells.	
Neointimal cells	Cells that arise by migration from media or adventitia into the intima. Neointimal cells may also arise by transdifferentiation of endothelial cells.		
Pericytes	Cells around small arteries and capillaries that lack a medial layer	PDGFRβ, NG2, RGS5, and smooth muscle alpha actin	56, 70, 111– 127
Adventitial fibroblasts	Cells external to the media. These cells typically lack smooth muscle markers but can acquire them and become neointimal cells or myofibroblasts.	FSP1, PDGFRα, periostin, Tcf21, and cell surface markers	128– 135
Adventitial stem cells		Sca1, KLF4, CD34, Gli1, and c-kit	136– 141
Myofibroblasts	The major cells seen in fibrosis, at least in some cases, arise from adventitial cells and express smooth muscle proteins.	RGS5 and smooth muscle alpha actin	142– 146

“Mural cells” is the current term for the mesodermal cells that provide a coat around endothelial tubes. At different times, these cells have been called fibroblasts or smooth muscle cells. The mature mural cell coats may be as thin as one cell layer or composed of many layers of smooth muscle cells separated by layers of elastin. The cells comprising single-cell layers associated with capillaries and small arterial branches are called pericytes. These pericytes have less abundant actinomyosin than the actinomyosin-rich cells of thicker media. Markers used to define pericytes versus smooth muscle cells include NG2 and platelet-derived growth factor (PDGF) receptor beta. The relationship between pericytes and smooth muscle cells, other than the caliber of vessel served, is unclear. We do not know that one cell cannot change expression and become the other cell depending on the local environment. Similarly, most authors do not consider a myofibroblast to be a smooth muscle cell, presumably because the myofibroblast can revert to a fibroblast phenotype.

## Normal intima versus neointima

As we have just discussed, intimal cell masses form spontaneously at specific sites in the intima of humans and other large animals ^4, 43^. We will refer to this cell mass as the “normal intima” or “normal cellular intima”. Other cellular intimas, called “neointimas”, form as pathological responses to almost any form of arterial injury ^42^, including radiation, mechanical injury, immune responses, and, of course, accumulation of lipid in the atherosclerotic intima ^43^.

Both neointima and, presumably, normal cellular intimas are prone to lipid accumulation when animals are fed a high-fat diet. Kovanen and Tabas have suggested that the extracellular matrix of cellular intimas may have properties that retain and accumulate lipid ^147^. As already noted above, the recent observations that SMCs can differentiate into macrophages raise an entire new set of ideas about how intimal cells might predispose to lipid accumulation ^54, 148^. In summary, it seems likely that pre-existing cellular intima of spontaneous origin has properties that initiate atherosclerosis and perhaps determine the ultimate outcome of the lesions over the many decades of lesion progression in humans ^149^.

## Genetic versus epigenetic changes

Of course, neoplasia implies a genetic origin, a mutation as is believed to be the cause of uterine leiomyomas ^12, 13, 16, 20, 150– 158^. If uterine leiomyomas begin as mutations, why shouldn’t clonal masses in arteries also begin as mutations?

There are at least three ways Benditt’s clones might arise without the mutations implied by the neoplasia hypothesis. The first, as proposed at the end of this review, is isolation of some cells within the internal elastic lamina as the intima forms. The second is endothelial–mesenchymal transformation (EMT) as happens in cardiac valves ^159, 160^. The third is migration of rare cells from the media ^8, 50, 161^. None of these hypotheses explains a selective advantage needed for clonal development.

The development of these initial rare cells into a clone suggests that some property causes the clone to survive and grow. The initial cell of the clone might grow because of properties of the milieu between the endothelium and the internal elastic lamella or because of unique properties of the subset of endothelial cells overlying the nascent lesion ^162^. Alternatively, the clone might reflect epigenetic changes as have been reported for uterine leiomyoma ^163^. Cells that make up the smooth muscle coats of arteries from diverse embryological origins ^8, 58, 164^ may have epigenetic properties that confer genetic advantages. A relevant example of heritable changes associated with the origin of vascular smooth muscle may be the distinct properties of aortic cells derived from neural crest versus somitic mesoderm ^59, 165– 169^.

## Of mosaicism and clonality

As time passed and regardless of Benditt’s ideas about neoplasia, clonality in the vessel wall became well established. Twenty years after Benditt’s paper, Mikawa and Fischman used fluorescent viral tracing to show that the media of avian coronary arteries is formed by clones ^170^. The fact that Mikawa and Fischman’s clones were spiral structures compared with the small number of cells comprising the arterial media of mice may explain the failure, so far, to identify large patch sizes (that is, clones) in the media of mice labeled by the confetti method ^2, 3, 5, 9, 46, 171– 176^. Possibly, results will be different once bar code-tagged mice allow analysis of clonality as was done with zebrafish ^177^.

Regardless of the findings in mice, we do know that the human media is made up of clones (that is, patches of cells that arise from single cells) ^178^. Chung *et al*. were able to show that somatic mosaicism in the arterial wall was not limited to the plaque ^178^. Mosaic patches present in the media led Chung *et al*. to propose that the plaque might arise by the amplification of cells from pre-existing patches in the underlying media ^178^. As noted above, we now know that something like this happens in mice forming a neointima in response to injury ^3, 46, 50^.

**Figure 2.**
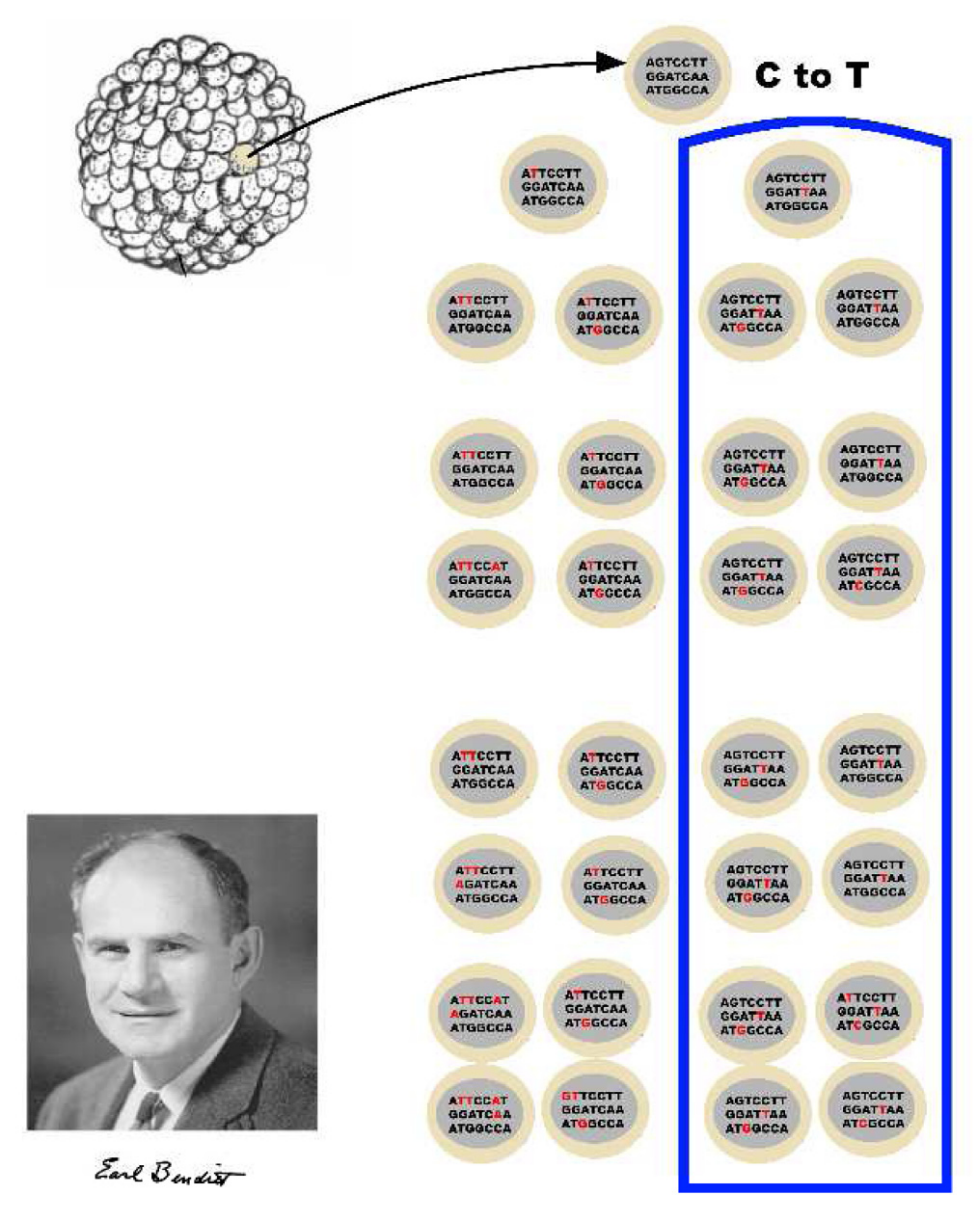
Clonality is normal. Clonal origins of normal tissues detected by the identification of mutations using DNA sequence analysis. Benditt might be surprised to learn that he, like all humans, was a collection of clones!

The idea that clonality is a normal part of tissue development is not unique to blood vessels. Highly accurate next-generation DNA sequencing is able to detect spontaneous mutations that occur as early as the blastula ^179^. Such mutations can be used to trace development because we can cluster cells in terms of subsequent mutations ^179, 180^ ( Figure 2). These methods permit the tracing of cell lineages of adult tissues as far back as events in gastrulation ^179^. Although we know that somatic mutations are a normal part of development, most such mutations are not functional and are not causal for clonal expansion ^179, 181^. Somatic mosaicism is nonetheless intriguing. Clonal segregation of mutations in the brain may provide important mechanistic clues to the function of different regions in the brain ^182– 184^. Presumably, such mutations exist in the human vessel wall as well.

The fact that plaques are clonal raises another possibility: functional mutations could arise in plaques as a result of, rather than as a cause of, clonal expansion. As a clone divides, replicating cells accumulate mutations that can provide a selective advantage over normal tissue, ultimately leading to a cancer ^181^. Consistent with the idea that clonal expansion in humans leads to DNA damage, there are reports of mutations and DNA damage in the plaque ^171, 185– 216^.

The diverse embryological origins of medial cells could provide a source of selective advantage for cells giving rise to an intimal clone. Embryological tracer studies of the media in mice have shown sources that range from mesectoderm derived from neural crest to mesoderm derived from the somites, the heart fields, mesothelium, and EMT ^8, 49, 59, 128, 164– 166, 169, 217– 229^. In some parts of the arterial tree, multiple sources may be represented in a single part of the vessel wall. Perhaps medial cells derived from diverse origins have selective advantages for growth in the intima.

Finally, a recent study suggests that clonal expansion could be a cause of plaque rupture. Wang *et al*. ^149, 230^ demonstrated telomere shortening in cells of the plaque and suggested that the plaque cells forming the fibrous cap may, as a result of many replications, undergo cell senescence. They suggest that cell death in the cap eventually leads to plaque rupture ^149^.

## Clonality in the immune system

Surprisingly, a recent report shows that clonality of leukopoietic lineages in bone marrow is relevant to the final events in atherosclerosis ^195^. Clonal diversity in the bone marrow decreases as we age while advantageous mutations undergo clonal selection ^231^. Presumably, this is due to mutations in a highly replicative tissue combined with replicative senescence ^231^. A mutation in the gene for the epigenetic modifier enzyme Tet2 promoted expansion of the mutant cells. One result of this is that leukemias in old age arise from a small number of surviving clones.

Fuster *et al*. showed that these changes in bone marrow lineages correlate highly with frequency of atherosclerotic clinical events ^195^. The authors went on to show that the expansion of Tet2-mutant cells in atherosclerosis-prone mice accelerated lesion formation. They proposed that age-related increases in death from coronary artery disease reflect the effects of inflammation attributable to this mutation in plaque macrophages derived from the mutated myeloid precursors.

Because T- and B-cell clonal selection occurs early in life, plaque lymphocytes may not be affected by the Tet2 mutation. In any case, because Benditt showed that the clonal cell type in lesions was a smooth muscle, it is not likely that lymphocyte clonality accounts for plaque monoclonality. Moreover, studies by Hansson *et al*. of plaque T cells showed oligoclonality rather than monoclonality ^232^.

## “What is a ‘cell type’?”

**Figure 3.**
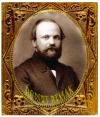
Omnis cellula e cellula. Robert Remak’s discovery of the modern concept of cell lineage is often falsely credited to Rudolph Virchow because Remak, as a Jew, had troubles getting published in mid-19th century Berlin ^237^.

**Figure 4.**
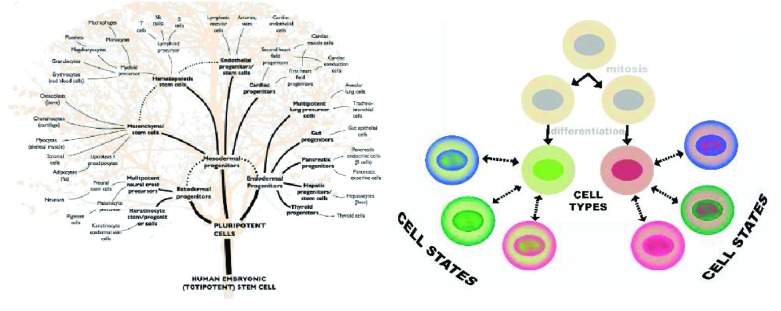
Cell type and cell phenotype are not necessarily the same thing. Cells in a lineage can change phenotype in a process called “phenotypic modulation”. Recent data combining bar codes with single-cell RNA analysis show that cells can even transdifferentiate across traditional tree-like boundaries.

As suggested above, the importance of clonality is tied to our concepts of cell type. In the 1850s, Robert Remak ( Figure 3) observed red blood cells in chicken embryos. These cells differentiated from cell precursors through various stages of cell division. His concept, our modern idea of cell lineage, was put into Latin as “ *omnis cellula e cellula*” (all cells come from cells) and became one of the central tenets of Virchow’s “Cellular pathology” text in 1856. Remak’s concept merged into the theory that cell type is determined by heritable changes that survive mitosis ( Figure 4). The genetic concept of cell linage became central to the dogma that the origins of a metastasis could be defined by its resemblance to a normal tissue, an idea supported by the “unitarian hypothesis” of Maximov ^233, 234^. The idea became set in the modern biology canon in 2002 when Brenner, Horvitz, and Sulston won the Nobel Prize for tracing the full developmental lineage of all of the somatic cells, 959 for males and 1,031 in females, in the nematode *Caenorhabditis elegans* ^235^.

**Figure 5.**
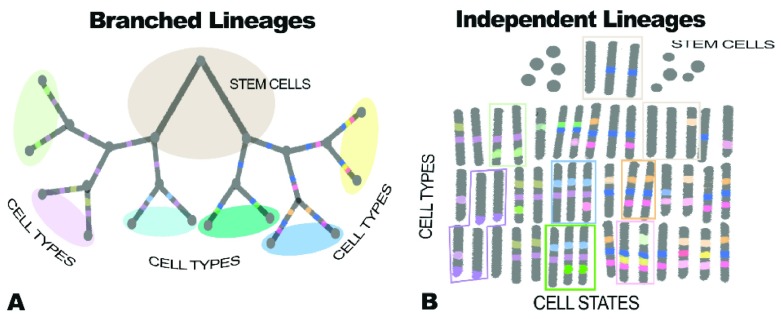
Branched lineages ( **A**) versus independent lineages with no branching ( **B**). These two figures show very different ideas about how cells differentiate. In A, cells differentiate more or less the way species form (that is, in a branched tree arising genetically). In B cells, the model is very flexible, with very similar cell types, marked here by green and red rectangles, arising by the coordinated expression of different sets of genes under epigenetic control. Mathematical modeling using both marker genes and gene clusters shows that traditional branched tree (that, is branched lineage) cells can also switch phenotypes even between branches of the tree ^236^. A formal way of thinking of this is that, rather than cell types, cells exist on the surface of a three-dimensional landscape as proposed by Waddington 60 years ago ^238^. In this view, cells that are very much alike exist in valleys of coexpression as shown in part B of this figure and discussed by Trapnell ^239^. Single-cell analysis of expression, combined with tools that can trace cell lineages in whole animals, now allows us to distinguish between branched and independent cell lineages as discussed in Figure 6.

Over 150 years after the publication of “Cellular pathology”, Remak’s concept is now challenged because of a combination of new data in epigenetics, analyses of expressomes using single-cell RNA analysis, lineage analyses using spontaneous mutations or bar coding, and network analysis using Bayesian networks and cluster analysis ^236^. We now know not only that cell types can branch in a tree-like fashion but also that, even independent of neoplasia, cells can jump between branches ^177^ ( Figure 5).

It is important to understand that Remak and Virchow developed the ideas of cell lineage before Mendel. Darwinian evolution, from its onset, has been understood to reflect heritable changes in what we now call a genome. In contrast, as shown in Figure 5b, differentiation is not based on mutations in DNA and thus lineage maps do not need to follow a Darwinian set of branched pathways. For example, in macrophage differentiation, macrophages resident in different tissues retain an epigenetic memory of origin sites ^240^, even when they move to new tissues. Moreover, macrophages themselves apparently can arise from very different lineages, including marrow-derived cells that originate from a subset of endothelium, tissue-resident macrophages that originate independently of the endothelium, and even SMCs ^46, 148, 162, 240– 247^.

## A new definition of cell type

The definition of cell types has traditionally been based on marker genes (that is, proteins found at high levels in a differentiated tissue). Examples include albumin for hepatocytes, immunoglobulins for B cells, insulin for pancreatic beta cells, vascular endothelial (VE) cadherin for endothelial cells, and cell type-specific muscle myosins or actins for heart and skeletal muscle. In the vascular media, cell type has been defined primarily by the location of cells and the high levels of expression of smooth muscle actin in cells organized as the tunica media ( Table 1). Smooth muscle actin, however, is not definitive because not all cells in the tunica media express this protein ^248^. Moreover, the expression of smooth muscle alpha actin is not restricted to smooth muscle. The gene is expressed in other muscle lineages as well as endothelial cells and even epithelial cells.

**Figure 6.**
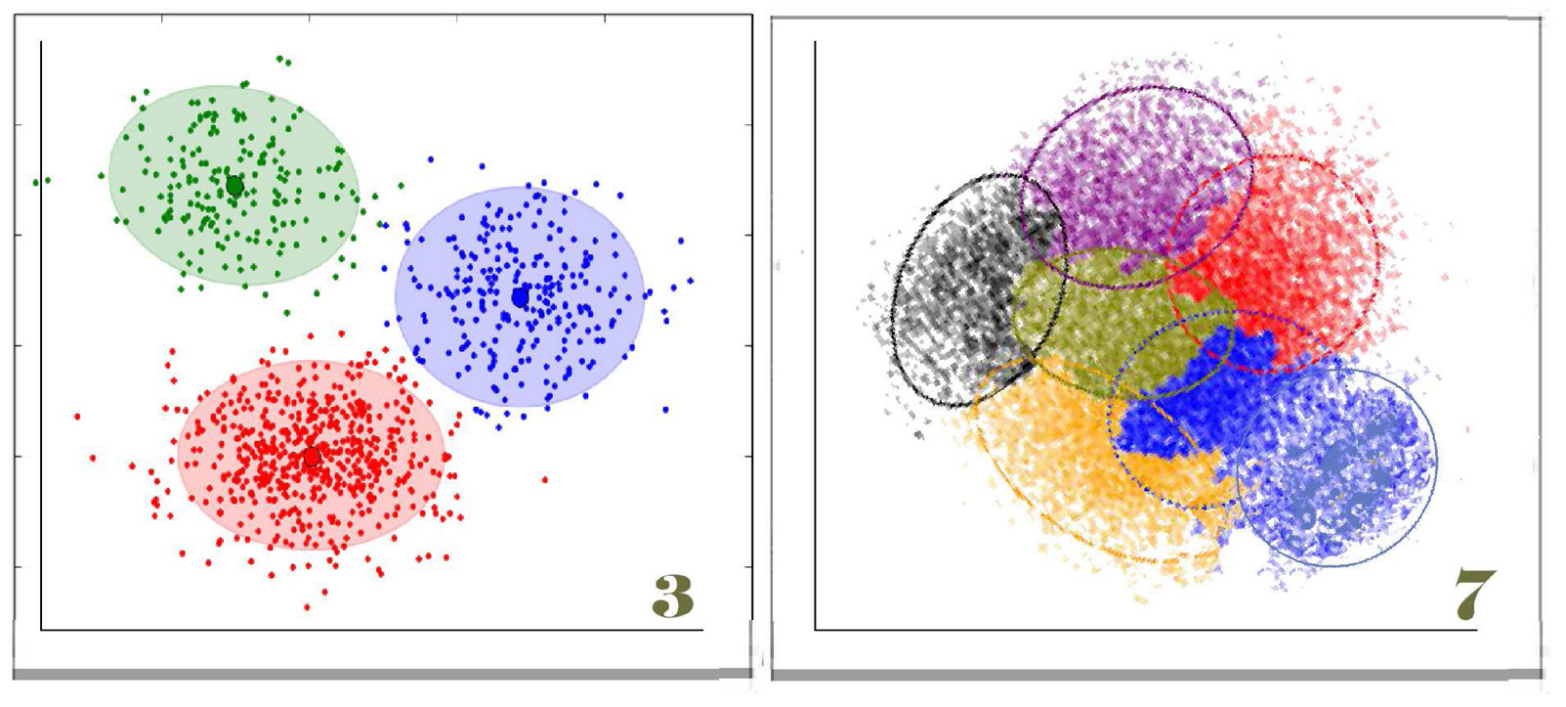
Defining cell type by cluster: three types and seven types? Clustering cells by analysis of their mRNAs, or any other sort of single-cell data, is a way of arranging cells independently of assumptions about any structure other than similarity in different data sets. For example, clustering is independent of assumptions about lineage as shown in Figure 5A. If you have samples of mural cells from 100 aortas of different mice, you could ask how many cell types or groups of different cells make up the aortic media. Obviously, the clusters will include marker genes, but, because the number of genes sampled is large, the identity of each cluster as a “cell type” may or may not depend on that marker. Thus, a cell lacking the expression of a marker gene (for example, smooth muscle actin) might still be defined as a cell type based on the mean values of its other transcripts. Defining a cell type by cluster analysis has three major advantages over the use of marker genes. First, because the cluster is defined as the mean level of mRNA of the set of sampled genes, the noise level arising from measurement errors is much lower than a definition based on only a single value. Second, the cluster data can be combined with other data (for example, variations in genetic sequence) to determine pathways that control the cell’s phenotypes. Third, existing knowledge of expression pathways can be used to imply the mechanism providing overall control of the cell type. Cell type analysis defined by clustering can also be combined with lineage analysis using bar coding methods, analysis of the mutations that occur in the generations after zygosis (as in Figure 2), reporter genes that give cells different colors, or reporter genes that reflect the expression of particular genes at earlier stages of development. The result is that we can distinguish between cell types as defined by a cell’s ancestry and the cell’s phenotype at a specific time. The two cluster diagrams in this figure illustrate a need for caution. Even when, as on the left, the three clusters appear clear, the graphic image may reflect biases by the investigator. Clustering algorithms usually assume that the biologist wishes to see the number of clusters that offer the greatest separation between clusters. Programs either begin with a random guess about the number of clusters or use the number suggested by the investigators. The algorithms then move individual “genes” or other nodes between clusters to find an optimum that minimizes variability within clusters while maximizing variability between clusters. As shown in the diagram to the right with seven clusters, it is also possible that clusters overlap. Discussion based in part on http://www.statsoft.com/Textbook/Cluster-Analysis.

A new approach may be to define a cell type by transcription analysis based on sets of coregulated genes. These sets are called “clusters” ^249– 251^. Understanding how we can define cell types by “clustering” levels of RNA expression ( Figure 6) requires that we consider how transcripts are measured. The accuracy of individual transcript levels may be less important than the numbers of transcripts measured in an assay. This is fortunate because, unless RNA levels are measured by quantitative polymerase chain reaction, efforts to determine the quantities of the 10,000 or more mRNA species in an individual cell type are fraught with error. These errors can be minimized in a number of ways, including using many probes for each putative RNA in a hybridization analysis, increasing the number of sequences identified by spending more money on sequence analysis, or clustering sets of coregulated genes ^250– 253^. Because there are many genes in a set, a mean value of expression for all genes in a cluster (that is, its center of gravity) has less error than the error of any one gene ^249– 253^. As a result, cluster analysis can be used to define cell types independent of any marker gene.

Of course, pathways are the ultimate definition of cell type and cell state. Covariation of the quantity of different RNAs ( Figure 6) has been used for at least two decades to identify pathways. These pathways can be connected to causal Bayesian networks by identifying the sites of variation in gene sequence that control variation in expression ^254– 258^. These approaches have been used to define cell types in terms of a cluster of genes rather than individual marker genes ^259, 260^ ( Figure 4– Figure 6).

Recently, in the vessel wall, cluster analysis has been used to define cell types using single-cell RNA analysis. Carried out over a time course and combined with the introduction of artificial DNA bar codes, this can be used to map the lineage of essentially every cell as an embryo develops ^261– 263^. The method has even been used to identify unexpected lineages when cloned stem cells were induced to develop into skeletal muscle ^239^. Betsholtz and collaborators used cluster analysis to identify four different types of pericytes coating brain capillaries in the vessel wall ^56^. Pericytes are smooth muscle actin-expressing cells that form the thin layer of cells surrounding the endothelium of capillaries ( Table 1). One of these pericyte types, with a distinct location in the capillary bed, lacked smooth muscle alpha actin, the canonical marker for SMCs ^56^.

Once defined, a cluster can be used to simplify transcription analysis of all the genes expressed in a cell. Moreover, since a few genes can be used to determine the expression of genes belonging to coregulated clusters ^253^, accuracy of a cluster can be quite high. For example, hybridization chips that use fewer than 1,000 carefully curated probes to represent the entire transcriptome were recently designed ^253^. These “L1000” chip probe sets reduce the cost of analysis of a transcript profile to less than $10. Because these chips are inexpensive and not limited by counting errors associated with RNA sequencing, very large numbers of cell types or cell states can be cheaply defined and stored in a common database. Currently, the consortium behind L1000 has analyzed 1.3 million samples representing different cell states or cell types ^253^. The low cost of L1000 chips may make the identification of cell types and cell states very inexpensive. In summary, rather than single genes, clusters may provide a better way to define cell type ^56^.

One concern is that the statistical construct of clusters may mean that we need to live with the idea that cell types overlap. Of course, that overlap may be the true meaning of cell type.

## A semantic issue: “phenotypic modulation” versus “cell state”

A more general term than “phenotypic modulation” may be “change in cell state”. Whereas loss of cell differentiation in response to injury is common to most tissues responding to injury, the term “phenotypic modulation” is peculiar to vascular biology. The term had its origin in the early days of SMC culture when Julie and Gordon Campbell observed that cultured SMCs, like all other cells adapted to grow in culture, lost their differentiated phenotype. Cultured SMCs switched from a “contractile” *in vivo* to a “synthetic” phenotype adapted to growth in culture. The Campbells proposed that the loss of the contractile proteins, especially smooth muscle alpha actin, was central to the migration of medial cells and proliferation in the intima to form a neointima ^264, 265^. In subsequent work by Feil *et al*. ^62^, Owens *et al*., and others ^46, 53, 62, 63, 266– 275^, much of the signaling process controlling this switch has been worked out. The signaling process involves transposition of a transcription factor from the promotor driving actin to a promoter driving genes required for cell proliferation.

As illustrated in Figure 4, changes in cell state occur in most cell types, including post-mitotic, terminally differentiated neurons that respond to a severed axon by switching to a phenotype that regenerates the injured axon ^276^. As an extreme example, fibroblasts can be induced to differentiate into skeletal muscle by overexpression of the transcription factor myoD. The skeletal muscle type, however, is reversible if the cells are demethylated by introduction of bromodeoxyuridine (BUdR). When BUdR is removed, the cells redifferentiate presumably because of the persistence of epigenetic changes ^277^. In this case, we might argue that the skeletal muscle cell type persisted even when the markers of skeletal muscle phenotype were gone.

The semantic issue may be whether one means “phenotypic modulation” to distinguish between reversible changes in phenotype (that is, cell states) or whether the term implies the more permanent properties that identify cell types. For example, most differentiated cell types can respond to tissue injury by adopting motile or replicative phenotypes. When the tissue has regenerated, the cells revert to their resting state. Even post-mitotic, terminally differentiated neurons are capable of undergoing chromatolysis. Chromatolysis is a massive increase in the proportion of DNA unfolded and involved in transcription as is needed to regenerate an injured axon ^276^. Similarly, hepatocytes, skeletal muscle, renal tubular epithelium, and so on all undergo reversible changes in cell state when induced to regenerate after a wound.

As an example closer to vessel wall biology, the “myofibroblast” is derived from fibroblasts. The resting fibroblast lacks smooth muscle alpha actin or other markers of the smooth muscle type ^278^. Following stimuli by certain cytokines, fibroblasts transform into a phenotype characteristic of a healing wound with an abundance of synthetic endoplasmic reticulum to make connective tissue and actin-rich microfibrils to promote wound closure. However, as the wound progresses, the myofibroblasts cease making matrix and lose their actin-rich content. They again appear as fibroblasts. Similarly, even if we define the clonal cells in a plaque as belonging to a smooth muscle “cell type”, it is not at all surprising that we can find SMCs in a proliferative or other state as required by the local environment ^46^.

Whatever terminology we use, it is important to realize that Remak’s concept of cell lineage may no longer be correct ( Figure 5). Wagner *et al*., combining single-cell RNA analysis with bar codes, found lineages that cross between the expected branches, implying that “cell types” can give rise to cells that otherwise would be assumed not to exist because of membership in a different lineage ^177^. Wagner *et al*. concluded that “the ability of embryonic clones to undergo dramatic converging/diverging behaviors thus underscores a continued need for independent measurements of both cell state and lineage in the mapping of cell fate hierarchies” ^177^. An important pathological example of a switch across lineages includes papers showing that glioblastoma stem cells, presumably of neural crest origin, are able to express both the endothelial phenotype and the SMC/pericyte phenotype ^279^.

As already discussed, recent reports have described a change in smooth muscle phenotype that may even comprise a change in cell type. We already know that macrophages derived from cells in different organs retain epigenetic changes from that origin even when challenged with cytokines characteristic of different resident macrophage cell states ^243, 244, 280^. However, it now appears that intimal cells that derive from medial smooth muscle can acquire macrophage properties ^52– 55, 148^. The lineage issue becomes even more problematic with the recent reports that all macrophage and endothelial cells derive from a common precursor, the “erythro-myeloid progenitor” (EMP). In view of the ability of cells to cross traditional lineage barriers, it would be interesting to determine whether RNA expression data from these macrophage-like cells cluster with other tissue-derived macrophages, with macrophages derived from the bone marrow ^240, 243, 244, 280^, or perhaps even with endothelial cells undergoing endothelial–mesenchymal transformation.

In summary, we do not know whether phenotypic modulation allows an SMC to revert to a fully contractile phenotype. Put into conventional terms, perhaps intimal cells are their own “cell type”?

## Origins and identity of “mural cells”: another semantic issue

The term “mural cell” is used in vascular biology for the connective tissue cells that form the wall around endothelial tubes. Because the term “mural cell” is based in morphology, the term can replace “vascular smooth muscle cell” without defining a cell type by lineage and without making assumptions based on the expression of a single gene ( Table 1).

**Figure 7.**
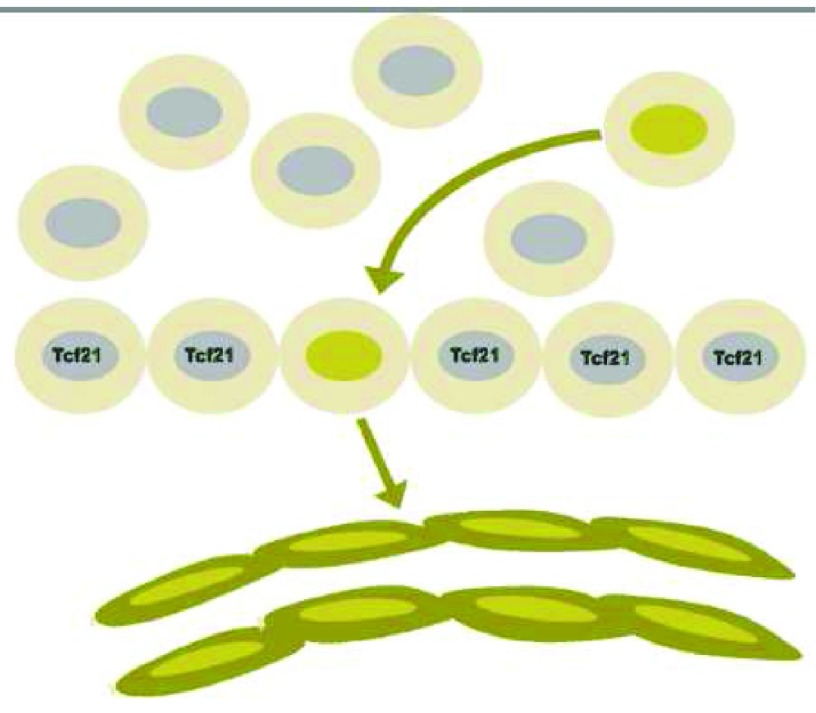
Endothelial cell precursors can be identified by lineage tracing within the proepicardial organ before cells from that precursor migrate to form the epicardium. Cells marked by the expression of the transcription factor Tcf21, in contrast, give rise to the mesenchyme of the heart, including both smooth muscle cells and fibroblasts.

We need to understand how the formation of the endothelium determines the origins of mural cells ( Figure 7). The angioblasts arise during gastrulation. Angioblasts are the progenitors of endothelium, monocytes, lymphocytes, and all white cells and red cells. Angioblasts, or perhaps the more primitive EMPs ^162^, form endothelial cells as well as the precursors of tissue macrophages, including glial cells of the brain ^243^. The endothelial cells form themselves into tubes, and these tubes join up and branch to form a capillary plexus. The plexus is a chaotic network that remodels into the orderly branched vascular tree with arteries, capillaries, and veins. Tissue macrophages, called histocytes, arise by migration of their precursors from the EMP. Monocytes arise from EMPs that become part of the endothelium and give rise to precursors that detach and populate the bone marrow and other hematopoietic sites.

The branched endothelial tubes acquire coats of mural cells derived from various local forms of mesenchyme. The recruitment of mural cells does not occur until the branched tree of arteries and branches reaching all parts of the growing embryo have been formed. There is no evidence that marrow-derived cells give rise to precursors of mural cells.

Mesodermal sources of progenitors for mural cells are diverse. The sources range from the mesectoderm of the neural crest, the mesothelium of the thoracic and abdominal cavity, the heart fields, the endocardium, the epicardium, the metanephros, and the somites. In some places, mural cells may even develop by transdifferentiation of the endothelium ^49, 59, 166, 222^.

Not all mural cells go on to express smooth muscle alpha actin. In chickens, the media of the thoracic aorta contains two ultrastructurally distinct cell types, one cell having the characteristic filament-rich cytoplasm of an SMC and the other the ultrastructure of a fibroblast ^248^. Embryological studies in chickens and mice further suggest that this morphology may represent unique origins from the somatic mesoderm versus the mesectoderm formed from neural crest. Similarly, mural cells surrounding certain capillaries in the brain fail to express smooth muscle markers ^56^.

Given origins in distinct regions of the mesoderm, there is no reason to assume that the resulting cell populations would exist in a single-cell state, even if all of these cells were defined as being of one SMC type. Mural cells with different origins show distinct proliferative properties and biosynthetic profiles, even in cell culture ^64, 169, 281– 285^. In mice, Roostalu and Wong have recently shown distinct lineages within the media itself, including a distinct population of mural cells arising during embryonic development that is maintained postnatally at arterial branch sites ^226^. Finally, similar studies show that multiple embryonic sources within the developing mouse heart contribute to the cells that become the medial smooth muscle of the coronary arteries ^286^. As we will also discuss below, Tallquist *et al*. have shown that the epicardium gives rise to two distinct lineages: one that forms the media and another that forms the adventitia ^128^.

Finally, data from studies of differentiation of organ-specific epithelium suggest that the origins of different mural cells might affect the cell state of organ-specific endothelial cells ^287, 288^. Differentiation of the endoderm into organ-specific types of epithelium depends on the interaction of epithelial cells with organ-specific mural cells and their matrix ^289– 291^. By analogy, it seems possible that different mural cells and intimal cells control the phenotype of overlying endothelium. Conversely, differences in lineage within the endothelium might control the formation of intimal cell masses ^162^. Such cell type interaction-specific interactions between epithelium and mesenchyme might explain the localization of atherosclerosis at specific sites.

## Possible sources other than mural cells for origins of intimal cells

The media is not the only possible source for intimal cells. Studies using labeled bone marrow cells in mice and humans have purported to show that intimal SMCs in atherosclerotic plaques could be derived from circulating marrow-derived cells ^292, 293^. These studies, however, have not held up to more careful work ^8, 47, 294^.

**Figure 8.**
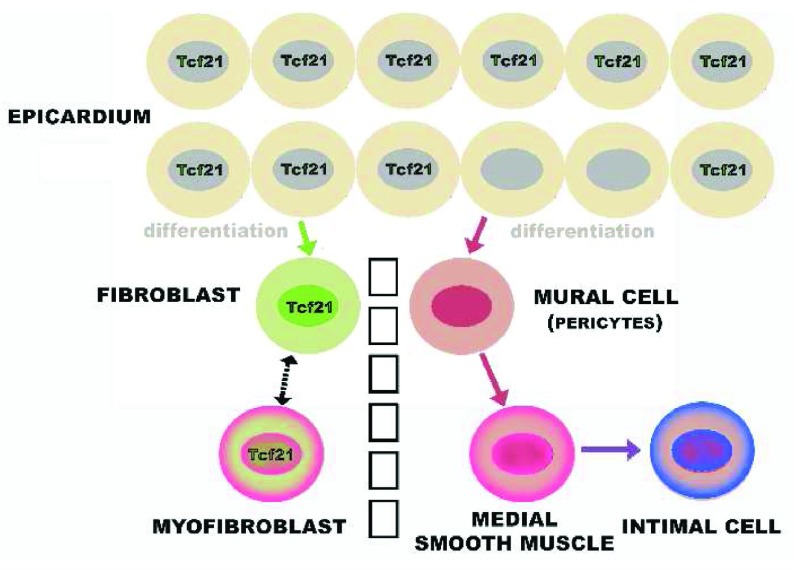
Layers of the artery wall. The intima is a layer of connective tissue located between the endothelium and a layer of elastin called the internal elastic lamina. The media is delimited by the dashed black lines representing the internal elastic lamina and the external elastic lamina (EEL). Only rare cells, including lymphocytes and smooth muscle cells, are seen in the normal intima of the small mammals usually used to study atherosclerosis. However, in humans, intimal cells accumulate spontaneously during normal development and appear as a clone in the atherosclerotic lesions of adult humans. Based on immunocytochemistry, these cells are usually considered to be smooth muscle cells. In fat-fed animals, including humans, lipid accumulates in the intima to form the characteristic fatty atherosclerotic lesion. The outer limit of the tunica media is also defined by a layer of elastin called the EEL. Extrinsic to the EEL is a poorly defined tissue that is part of the matrix surrounding not just blood vessels but the parenchymal cells that comprise organs. The part of this matrix close to the vessel wall is called the adventitia.

A more likely source is the adventitia. The adventitia of arteries is defined as those cells outside the external elastic lamina ( Figure 8). This layer contains endothelial cells, neurons, blood-derived macrophages, lymphocytes, mast cells, and fibroblasts. Recent studies show that adventitial fibroblasts include cells identified as stem-like by the expression of markers like c-kit and sca1 ^224^. Cell tracing studies show that adventitial cells can cross the media of injured vessels to form a neointima ^136, 295, 296^. Moreover, transplantation studies *in vivo* show that adventitial stem cells applied to the outside of an injured vessel can migrate across the media and form an intima ^295^.

Adventitial fibroblasts are also of interest because of their relationship to fibrotic responses. Outside the vessel wall, adventitial cells respond to injury by the synthesis of high levels of smooth muscle actin, becoming the major cell type seen in fibrosis, the “myofibroblast” described above ^297, 298^. Myofibroblasts characterize scleroderma and may be derived from vessel wall cells ^142^. Myofibroblasts (that is, fibroblasts rich in smooth muscle actin) also characterize the mesenchyme of some tumors in a process called “desmoplasia” ^299– 301^. Curiously, no effort has been reported to use cluster analysis to compare myofibroblasts with intimal cells.

**Figure 9.**
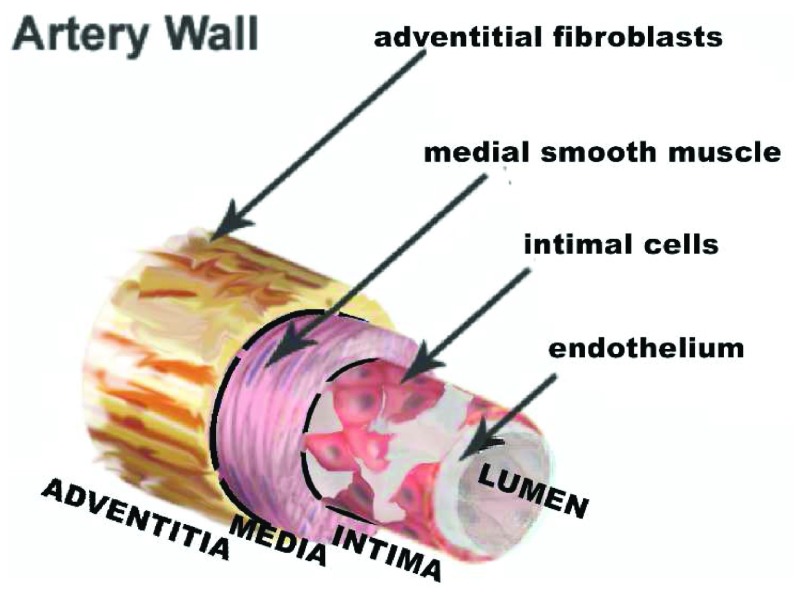
Dichotomous origins of medial smooth muscle and adventitial fibroblasts. Both arise from the epicardium, but the smooth muscle lineage requires first the loss of expression of Tcf21. Presumably, the intimal cells arise from the medial smooth muscle but, as discussed in the text, we cannot rule out origin from the adventitia, especially since adventitial fibroblasts are the source of smooth muscle actin-rich myofibroblasts seen in injured myocardium.

The origin of intimal cells from adventitial cells may imply that intimal cells are of a different cell type than medial cells. Tallquist *et al*. showed the adventitial cells of coronary arteries and the mural cells of the coronary artery media derive from distinctive precursors in the epicardium ( Figure 9) ^128^. This differentiation occurs when epicardial cells lose the expression of a transcription factor, Tcf21. The Tcf21-negative cells undergo epithelial–mesenchymal transformation, migrate to coat the nascent endothelial tubes, and form mural cells. These mural cells initially have the properties of pericytes and populate the entire coronary microvasculature ^302^. In contrast, the fibroblasts around these vessels, that is the adventitial cells, originate from the Tcf21-positive epicardial cells by migration ^128^. Presumably the adventitial cells express smooth muscle actin only during cardiac fibrosis when they become myofibroblasts. The observations of Tallquist *et al*. have not as yet been extended to other vascular beds ^128^. Much less is known about the origin of adventitial cells other than those in the heart ^137, 223, 224, 303^.

There is, finally, one additional possible source for intimal cells, the endothelium. Endothelial cells are capable of undergoing EMT ^228, 304– 306^. EMT has been intensively studied in the formation of the cells making up the cardiac valves ^129, 160, 304, 307^. In addition, Karsan *et al*. used the Tie1 promoter to identify an endothelial-like cell that is present in the vasculature of developing murine vessels. This labeled cell differentiates into smooth muscle. Endothelial cells that also showed the canonical VE cadherin marker, however, did not differentiate into smooth muscle ^308^. It is intriguing to wonder if the normal intima might originate in such a peculiar precursor cell. The origin of the intimal cells of the human coronary artery from a human cell equivalent to the Tcf21-positive epicardial cells of Tallquist *et al*. or by EMT would provide an entirely new perspective on the meaning of clonality.

## An intimal hypothesis based on fenestrae

**Figure 10.**
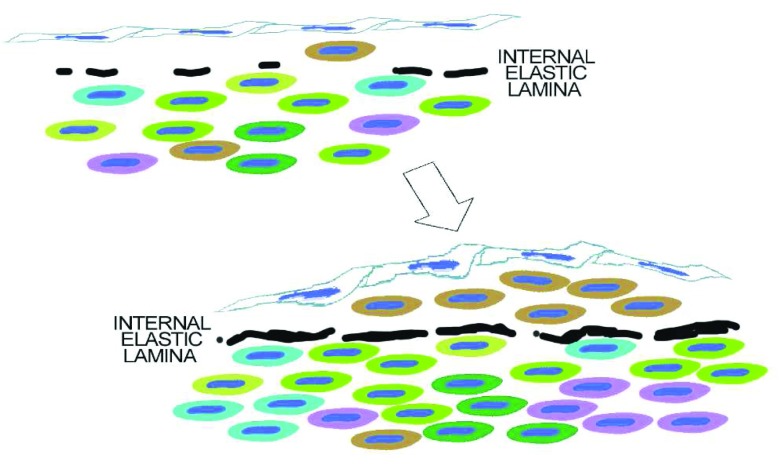
The simplest hypothesis. As discussed in the text, intimal smooth muscle cells in adult human atherosclerosis are clonal. Although we do not know that this clone is derived from intimal clones present at birth, this is an obvious hypothesis. The next question might be how would normal intima develop as a clone? The simplest hypothesis is that some mural cells that coat the arterial endothelium get trapped within the forming internal elastica. Isolated from other mural cells, these intimal cells would develop a phenotype dependent on both their lineage and the conditions of being confined between the endothelium and the internal elastic lamina.

Figure 10 proposes perhaps the simplest model for plaque monoclonality. In this model, rare mural cells are trapped within the intima as the internal elastica forms and the fenestrae, holes in the internal elastic lamina, shrink ^309, 310^.

A simple alternative is that migration from the media is limited not only by the number and size of passageways across the internal elastic lamella but also by secretory and cytoskeletal changes required for transfenestral migration. Fetal properties that allow some medial cells to migrate may be developmental but could be reactivated in a subset of the medial SMCs when arterial injuries occur. A recent study by Lu *et al*. ^311^ showed examples of transfenestral SMC migration in wild-type arteries after carotid ligation and in transgenic mice with Mef2c deletion specifically in endothelial cells.

Finally, localization of intimal cells might be the result of stimuli coming from endothelial cells responding to flow ^312^ or from subsets of endothelial cells with their own lineages ^162^.

Figure 10 does not rule out the possibility that the clone selected for growth in the intima has special properties derived from its developmental lineage, acquired because of the environment of an injured media, or resulting from mutation.

## In the end, does intimal clonality matter?

We do not know whether the origin of a lesion in clones of intimal cells is important to the natural history of the plaques. The common belief is that atherosclerotic lesions develop selectively in areas where the arterial endothelium is exposed to turbulent blood flow ^312^. However, as already discussed, we know that the neointimas formed after injury predispose to atherosclerosis and that sites with similar rheology may or may not develop atherosclerosis. Therefore, it seems likely that spontaneous and intimal masses may be the initial cause of atherosclerosis in humans.

Moreover, sites that show spontaneous intimal thickening ^26, 313^ are also sites that manifest in later life as lesions that rupture, leading to occlusion and thromboembolic events. Perhaps localization of lesions at these sites is accelerated because of synthesis of matrix proteins that bind lipoproteins ^314^, because of pro-inflammatory interactions between endothelial cells and intimal cells ^51^, because of regional differences in Hox gene expression and nuclear factor-kappa B (NF-κB) activity which correlate with areas of lesion formation in aortas of atherosclerosis-susceptible mice ^315^, because of localized differences in the synthesis of elastin which eventually affect plaque stability ^316– 240^, because telomere shortening due to clonal amplification leads to senescence, cell death, and rupture of the fibrous cap ^149^, or even because of immunological properties of the macrophage phenotype derived from medial SMCs ^53, 320^.

## Summary

### New frontiers that have emerged since Benditt’s original observation

Given all that we have learned from mice and from the success of lipid-lowering drugs, Benditt’s observation would be irrelevant today if we did not know, as in Figure 1, that the intimal cell mass precedes the development of the fatty lesions. Benditt’s hypothesis would still be irrelevant unless we imagined that the natural history of the plaque over decades of plaque development could depend on the identity and properties of the clonal cell type making up the intimal cell masses.

Our review has tried to make seven points:

1. Benditt’s observations have held up, but his neoplastic hypothesis has not. Even though carcinogens can promote the growth of intimal cell masses ^21^, there is (as yet) no evidence that the clonal growth of the human plaque is due to a mutation. Perhaps such evidence will emerge with applications of next-generation sequencing to detect mutations in the plaque cells ^181^.
2. Intimal cell masses in human arteries precede atherosclerosis. However, we do not know that these masses are clonal. It is conceivable that clonality develops later as lesions develop as is seen in mice responding to arterial injury.
3. Intimal cells may determine where plaques develop. It is possible that intimal cells control the phenotype of overlying endothelium, including properties that attract leukocytes leading to the development of atherosclerotic lesions. Intimal cells may also provide an environment that accumulates toxic products of lipids.
4. Intimal cells have properties distinct from our traditional view of SMCs. We do not know whether these properties are reversible or represent a fixed change (that is, something that might be considered a “cell type”).
5. Cluster analysis, combined with lineage tracking, has opened a new frontier in defining cell types within the intima. It will be important to use these methods to define the cell types comprising the atherosclerotic clone.
6. The extent of intimal cell masses across the normal human arterial tree of children is not known. There has been no thorough study of the arterial intima in the arterial tree of human children or other large mammals. Such masses may also occur later in life and lead to the development of new atherosclerotic lesions as suggested by DeBakey and Glaeser ^29^.
7. Differences between models of atherosclerosis in mice versus the disease in humans may reflect the fact that mice lack intimal cells. Intimal cell masses, probably clonal in origin, may provide a soil for lesions that are distinct from those in the mouse model systems.
